# How the 2018 US Physical Activity Guidelines are a Call to Promote and Better Understand Acute Physical Activity for Cognitive Function Gains

**DOI:** 10.1007/s40279-019-01190-x

**Published:** 2019-09-18

**Authors:** Yu-Kai Chang, Kirk I. Erickson, Emmanuel Stamatakis, Tsung-Min Hung

**Affiliations:** 1 Department of Physical Education, National Taiwan Normal University, Taipei, Taiwan, ROC; 2 Institute for Research Excellence in Learning Science, National Taiwan Normal University, Taipei, Taiwan, ROC; 3 Department of Psychology, University of Pittsburgh, Pittsburgh, PA USA; 4 Discipline of Exercise Science, College of Science, Health, Engineering and Education, Murdoch University, Perth, WA Australia; 5 Charles Perkins Centre, Prevention Research Collaboration, School of Public Health, University of Sydney, Camperdown, NSW Australia

## Introduction

The new 2018 edition of the Physical Activity Guidelines for Americans (PAGA18) released by the U.S. Department of Health and Human Services [1] and directly informed by the 779-page *2018 Physical Activity Guidelines Advisory Committee Scientific Report* [2] will undoubtedly change how we promote and research physical activity (PA). For the first time, PAGA18 include new insights on the role of a single session of PA for cognitive function and brain health, suggesting that the scientific evidence supporting PA benefits on cognitive function and brain health has matured. In addition, considering the difficulty in initiating and adhering to a long-term exercise program, cognitive function benefits from a single bout may provide a new approach to promote exercise for people who are not ready yet to adopt and adhere to an ongoing habitual exercise routine.

## Current Recommendation: Acute Exercise and Cognitive Function

A single session of PA is known as an acute or single bout of PA [2, 3], and ranges from light to moderate intensity (e.g., walking) to maximal intensity training (e.g., sprinting) with a duration of a few seconds (e.g., sprint interval training) to several hours (e.g., a marathon). Cognitive function is the comprehensive term that describes the processes of acquiring and processing information involving but not limited to information processing, attention, intelligence, executive function, and memory [3]. These cognitive functions are primary components of quality of life, and their development (e.g., brain maturation and academic achievement) and decline/impairment (e.g., dementia and Alzheimer’s disease) are important across the lifespan.

PAGA18 [1] highlights the role of a single episode of moderate-to-vigorous PA as a facilitator of performance across several cognitive domains including executive function, processing speed, memory, attention, and academic achievement tests. The transient beneficial effects following a single bout of PA also affect crystalised intelligence in all periods of the lifespan with larger effect sizes in preadolescent children and older adults relative to other populations [2]. The PAGA18, along with the 2014 American College of Sports Medicine’s guidelines that also addressed the relationship between PA and cognitive function [4], provide a landmark for the prescriptions of a single bout of exercise to facilitate a variety of improvements in cognitive function.

## Optimizing Future Prescriptions of Acute Exercise: The 3W1H Framework

**Fig. 1:**
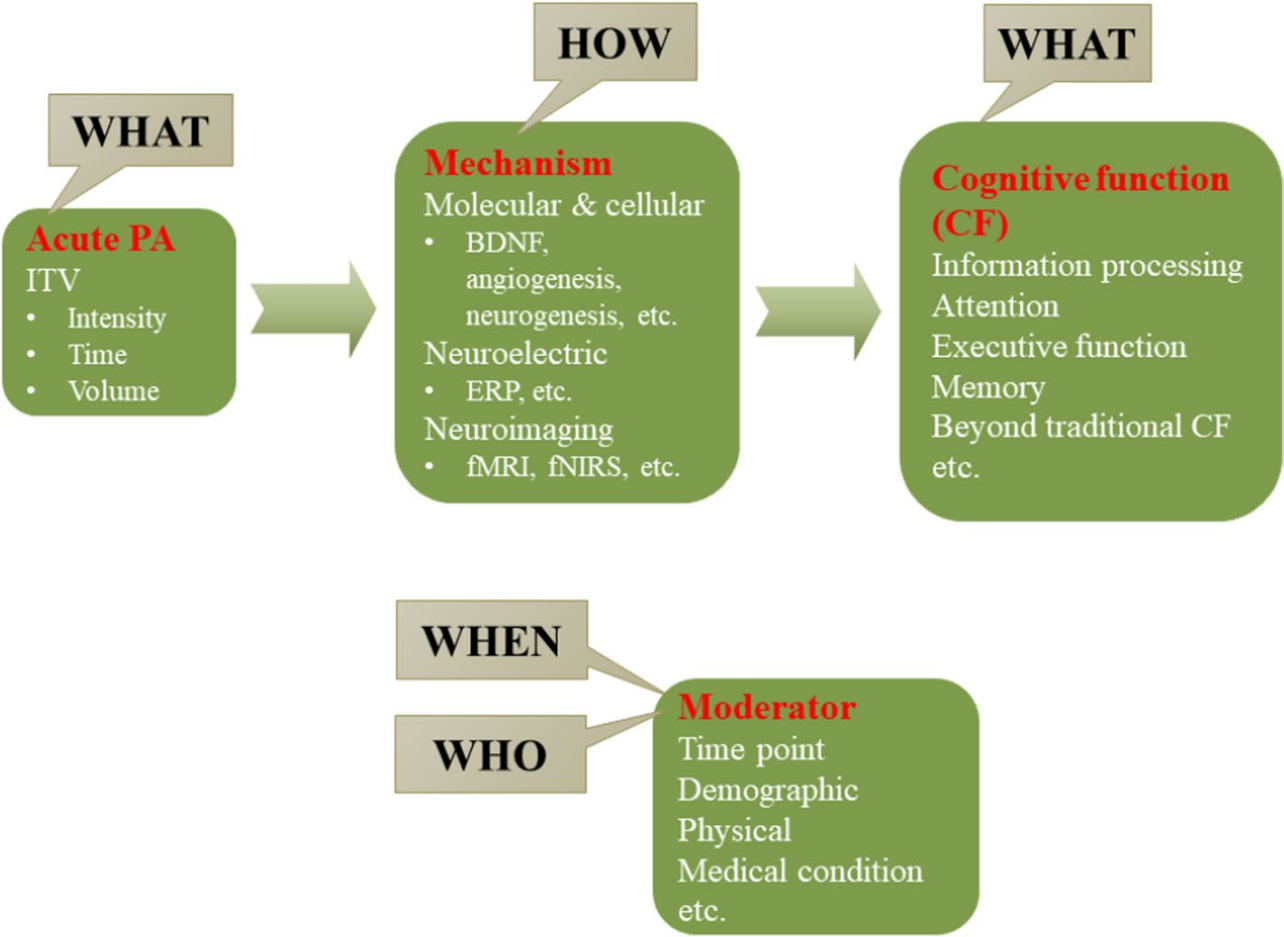
3W1H framework: future consideration for single bout of physical activity prescription on cognitive function. *PA* physical activity, *ITV* intensity, time, and volume, *BDNF* brain-derived neurotrophic factor, *ERP* event-related potential, *fMRI* functional magnetic resonance imaging, *fNIRS* functional near-infrared spectroscopy

Despite the evidence included in PAGA18, research on the effects of a single bout of PA on cognition remains sparse with clear gaps in understanding. Here, we propose a simple research framework, the 3W1H (i.e., what, who, when, and how) along with “ITV (i.e., intensity, time, and volume)”, to empirically test PA prescriptions that are specifically designed for improving cognitive function (Fig. 1).

### What?

The FITT-VP principle (frequency, intensity, time, type, volume, and progression), proposed by the ACSM [4], provides the essentials regarding “what” to tailor for optimal prescription, whereas the “ITV” PA dimensions are specifically linked to a single bout of PA. These guiding principles for exercise prescription would test the presence of a dose–response relationship between *x* and *y*. That is, how do cognitive outcomes change as a function of different doses of PA? Notably, the cognitive benefits appear to be greater for acute exercise sessions of light-to-moderate intensity than vigorous intensity [3].

The dose–response effects of acute exercise duration have been not been examined in detail [3, 5]. Chang et al. provide evidence that a single bout of exercise for 20–30 min is better compared to shorter (i.e., 10–20 min) and longer exercise durations (i.e., 45–55 min), indicating that the duration of an acute exercise session is an important factor when prescribing exercise as a means of influencing cognition [5, 6]. In addition, the positive effects of exercise are transient and may only be meaningful if they persist for more than just a few minutes [7, 8].

No study has examined total volume. For example, we do not know whether a single bout of PA of moderate intensity for 30 min is optimal, or whether there are different effects for a single continuous 30 min bout vs. 2 × 15 min bouts; or how a 15 min long bout of vigorous intensity compares with the roughly equivalent in volume of a 45 min light intensity bout.

“What” has also to do with the aspect of cognitive function: the effects of a single bout of PA on cognitive function appear to be dependent on the cognitive domain examined [3, 9], and therefore, the intensity and duration of the acute bout should be tailored to the cognitive domains being targeted. Moreover, the effects of acute PA on aspects of social cognition and emotion regulation that extend beyond more traditional views of cognitive function remain understudied and merit more attention. This is an important gap, because coping with emotions, empathy, and social cognition is an essential aspect of social beings [10, 11].

### Who?

There is insufficient meta-analytic evidence for answering the question of the characteristics of patient or person who will benefit the most from acute exercise in terms of demographic background (e.g., age, ethnicity, and socioeconomic status), physical status (e.g., obesity status and fitness level), genetic background, or medical history (e.g., diabetes and neurologic conditions).

### When?

The optimal time point for cognitive performance benefits following exercise has been examined systematically, with 11–20 min after the termination of exercise demonstrating larger effects than other durations [3]. Time of day might also be an important moderator, but only several studies have examined whether time of day of engaging in a single bout of PA (e.g., morning, mid-day, afternoon, and evening/night) influences cognitive benefits [3]. Interestingly, Wheeler and colleagues observed that acute exercise in the morning, with or without subsequent breaks, resulted in greater working memory and executive function benefits [12] as well as greater increases in cerebral blood flow [13] over 8 h in older adults. This result, although observational, suggests that acute exercise in the morning might influence the brain for a longer period and benefit cognitive and brain health throughout the day. These studies that examine acute exercise effects over the course of the day, especially with respect to other times of the day (e.g., mid-day), remain missing.

### How?

Large-scale randomized trials of acute exercise are rare. There is also a need for studies with relatively smaller sample sizes that are able to precisely control specific features of a single bout, and closely examine possible mechanisms from molecular and cellular levels as well as whole brain approaches using neuroelectric and neuroimaging techniques [14]. These interdisciplinary and often technologically demanding approaches that address simultaneously the “ITV” aspect of acute PA are often necessary for expanding understanding of the underlying mechanisms of the optimal exercise prescription for improving cognitive function.

## Conclusions

The evidence reviewed in PAGA18 regarding a single bout of PA for improving multiple aspects of cognitive function offers initial impetus to clinicians and policy makers to promote initiation of frequent acute PA that may hopefully turn into a lifelong habit for some. The 3W1H framework, along with ITV, provides some essential next steps to empirically determine the specific exercise characteristics that lead to optimal cognitive function gains across all age groups.
