# Correction to: How the 2018 US Physical Activity Guidelines are a Call to Promote and Better Understand Acute Physical Activity for Cognitive Function Gains

**DOI:** 10.1007/s40279-019-01225-3

**Published:** 2019-11-04

**Authors:** Yu-Kai Chang, Kirk I. Erickson, Emmanuel Stamatakis, Tsung-Min Hung

**Affiliations:** 1 Department of Physical Education, National Taiwan Normal University, Taipei, Taiwan, ROC; 2 Institute for Research Excellence in Learning Science, National Taiwan Normal University, Taipei, Taiwan, ROC; 3 Department of Psychology, University of Pittsburgh, Pittsburgh, PA USA; 4 Discipline of Exercise Science, College of Science, Health, Engineering and Education, Murdoch University, Perth, WA Australia; 5 Charles Perkins Centre, Prevention Research Collaboration, School of Public Health, University of Sydney, Camperdown, NSW Australia

## Correction to: Sports Medicine (2019) 49:1625–1627 10.1007/s40279-019-01190-x

The article How the 2018 US Physical Activity Guidelines are a Call to Promote and Better Understand Acute Physical Activity for Cognitive Function Gains, written by Yu-Kai Chang, Kirk I. Erickson, Emmanuel Stamatakis and Tsung-Min Hung, was originally published Online First without Open Access. After publication in volume 49, issue 11, pages 1625–1627 the author decided to opt for Open Choice and to make the article an Open Access publication. Therefore, the copyright of the article has been changed to © The Author(s) 2019 and the article is forthwith distributed under the terms of the Creative Commons Attribution 4.0 International License (http://creativecommons.org/licenses/by/4.0/), which permits use, duplication, adaptation, distribution and reproduction in any medium or format, as long as you give appropriate credit to the original author(s) and the source, provide a link to the Creative Commons license, and indicate if changes were made.

The original article has been corrected.

